# Dynamics and Thermodynamics of Transthyretin Association from Molecular Dynamics Simulations

**DOI:** 10.1155/2018/7480749

**Published:** 2018-06-05

**Authors:** Cedrix J. Dongmo Foumthuim, Alessandra Corazza, Rodolfo Berni, Gennaro Esposito, Federico Fogolari

**Affiliations:** ^1^Dipartimento di Area Medica, Università di Udine, Piazzale Kolbe 4, 33100 Udine, Italy; ^2^Istituto Nazionale Biostrutture e Biosistemi, Viale Medaglie d'Oro 305, 00136 Roma, Italy; ^3^Department of Chemical Sciences, Life Sciences and Environmental Sustainability, University of Parma, Parco Area delle Scienze 23/A, 43124 Parma, Italy; ^4^Dipartimento di Scienze Matematiche, Informatiche e Fisiche, Università di Udine, Via delle Scienze 206, 33100 Udine, Italy; ^5^Science and Math Division, New York University at Abu Dhabi, P.O. Box 129188, Abu Dhabi, UAE

## Abstract

Molecular dynamics simulations are used in this work to probe the structural stability and the dynamics of engineered mutants of transthyretin (TTR), i.e., the double mutant F87M/L110M (MT-TTR) and the triple mutant F87M/L110M/S117E (3M-TTR), in relation to wild-type. Free energy analysis from end-point simulations and statistical effective energy functions are used to analyze trajectories, revealing that mutations do not have major impact on protein structure but rather on protein association, shifting the equilibria towards dissociated species. The result is confirmed by the analysis of 3M-TTR which shows dissociation within the first 10 ns of the simulation, indicating that contacts are lost at the dimer-dimer interface, whereas dimers (formed by monomers which pair to form two extended *β*-sheets) appear fairly stable. Overall the simulations provide a detailed view of the dynamics and thermodynamics of wild-type and mutant transthyretins and a rationale of the observed effects.

## 1. Introduction

### 1.1. TTR: Origin and Function

**Figure 1:**
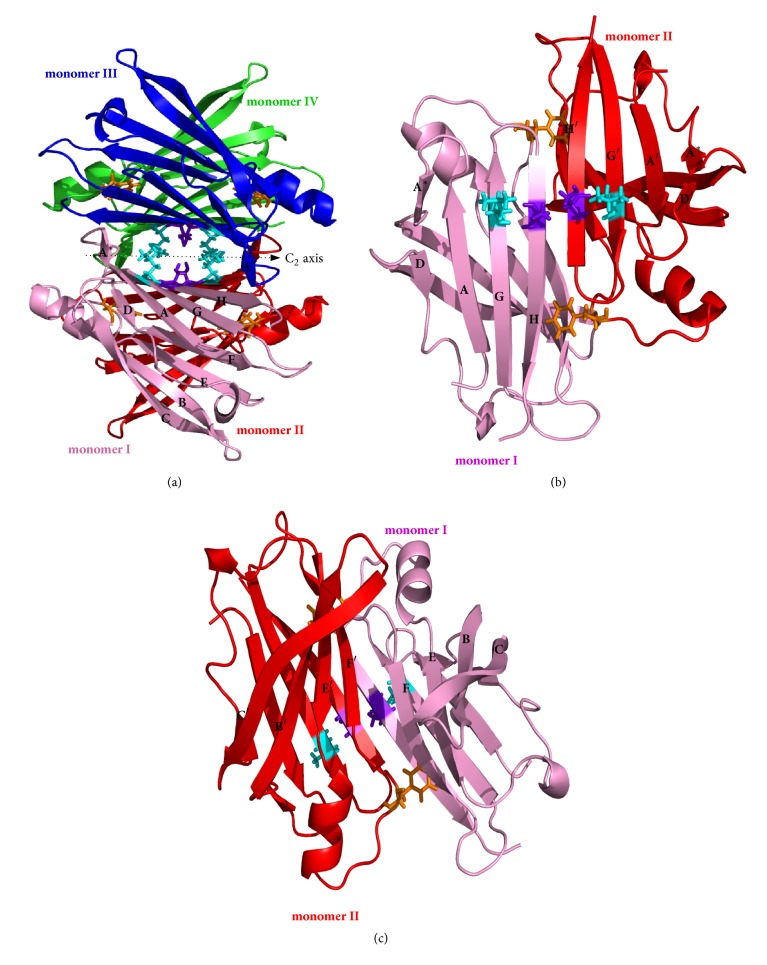
Cartoon view of tetrameric (a) and dimeric ((b) and (c)) assemblies of WT-TTR. Residues involved in the mutation (F87 (orange), L110 (cyan), and S117 (purple-blue)) are shown explicitly (stick representation) as well as the label of different *β*-stranded regions schematised in Figure 2, PDB id 1F41 [9].

Formerly known as prealbumin, transthyretin (TTR) is a 55 kDa globular oligomeric protein made up by four identical monomeric units (I-IV, see Figure 1(a)) each composed of 127 amino acid residues. Before being released into plasma, TTR is principally produced in liver, choroids plexus, and retina [1–4]. It is mainly a carrier protein that binds to retinol binding protein (RBP) to transport vitamin A in plasma and represents the leading transporter of thyroxine (T4) in cerebrospinal fluid (CSF) [5–7].

### 1.2. TTR: Structure

**Figure 2:**
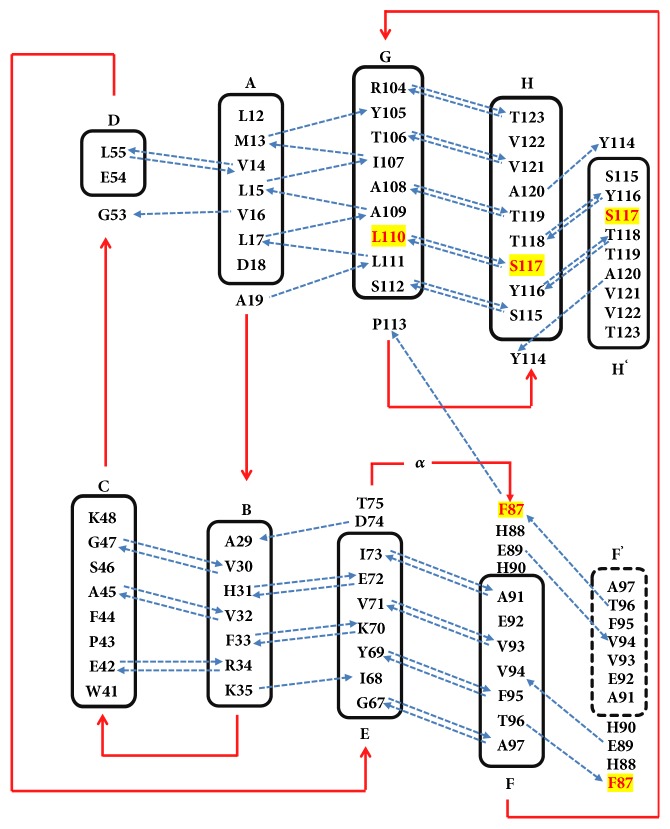
Topology arrangement of main chain-main chain hydrogen bonds (blue lines) within wild-type monomeric subunits and at monomer-monomer interfaces H-H' (Figure 1(b)) and F-F' (Figure 1(c)). Letters A to H designate the *β*-strands in one monomer while H' and F' belong to the other monomer of the same dimer. Red arrows indicate the connection between strands and show the loop regions and blue ones go from donor (NH) to acceptor (OC). Coloured residues (yellow) are the ones involved in the mutation and *α* is the helical region between strands E and F.

The 3D-conformation of all TTR-tetramers displays high degree of symmetry as shown at high resolution by Blake and coworkers [8] and Hörnberg et al. [9]. TTR is an overall *β*-sheet protein with a small *α*-helix domain between strands E and F (see Figure 1(a)). Its structure is highly ordered with a flexible N-terminal region that could not be resolved in many crystallographic studies. In each TTR monomer, about 45 % of residues are arranged in a sandwich immunoglobulin-like topology made up by two four antiparallel-strand *β*-sheets; the inner sheet DAGH is opposed to the outer sheet CBEF. Its secondary structure also exhibits a short *β*-strand portion A*∗* (see Figure 1) which is antiparallel (folded back) to strand A through a *π*-turn (i+5) and which is involved in dimer-dimer contact [10]. The dimer of monomers I and II (Figures 1(b) and 1(c)), also known as primary dimer, is the crystallographic asymmetric unit structurally symmetric to the dimer of monomers III-IV. It is stabilized by a network of six main chain hydrogen bond interactions established by three pairs of residues (A120(NH)⇆(OC)Y114, T118(NH)⇆(OC)Y116, Y116(NH)⇆(OC)T118) at the HH' interface (Figure 2). Monomer I inner sheet DAGH forms with its homologue H'G'A'D' in monomer II a kind of symmetric antiparallel pseudo-continuous eight-strand *β*-sheet centered on HH' (DAGH-H'G'A'D'), Figure 1(b) [10, 11]. At the opposite side, the pseudo-continuity in outer sheets CBEF-F'E'B'C'(Figure 1(c)) is rather loose and only 4 backbone hydrogen bonds (F87(CO)⇆(HN)T96 and E89(NH)⇆(OC)V94) are established (Figure 2). While dimers are mainly kept together by hydrogen bonds at monomer-monomer interface, tetramers become the dimers of dimers essentially stabilized by hydrophobic contacts established within neighbouring subunits at the dimer-dimer interface via AB-GH loop interactions involving mainly residues L17, A19, V20, L110, P113, T119, and V121.

### 1.3. TTR-Related Amyloidosis

TTR-related amyloidosis can be inherited in the case of genetic mutations or can be nonhereditary when it is due to wild-type (WT-TTR) [12]. WT-TTR amyloidosis also termed senile systemic amyloidosis (SSA) is the prevalent form of TTR amyloidosis which principally affects the heart. It develops with ageing and requires TTR tetramer dissociation and partial unfolding. Mutation-induced TTR amyloidosis is instead associated with familial amyloid polyneuropathy (FAP) which impacts essentially on peripheral nervous system and familial amyloid cardiomyopathy (FAC) with broad implication on heart [7, 12]. The interconnection between protein structure and stability and its capability to form amyloid fibrils have motivated several structural studies. To date, about 80 point mutations have been correlated to human inherited amyloidosis [13].

### 1.4. Designed Mutants

WT-TTR displays a very stable tetrameric molecular assembly. It is established that its structure is resistant to dissociation at physiological concentrations within the pH range 5-7 [14]. Several amyloid fibril formation models have been proposed and reviewed [15–17]. However, there is almost a consensus that the common mechanism involves the dissociation of the native tetrameric TTR into unstable but folded monomers, followed by local unfolding of the latter into multiple nonnative amyloidogenic intermediate states that self-assemble in solution [2, 6, 15–18]. Furthermore, evidence has been provided pointing to the dissociation of the native tetramer as the rate-limiting step towards aggregation [5, 19–22].

Therefore knowledge of the dynamics and thermodynamics of dissociation of TTR native structure is an important issue as tetramer dissociation, monomer misfolding, and self-assembly of amyloidogenic monomers into amyloid and other aggregate morphologies are known to be linked to several human degenerative diseases. Specifically, TTR represents one of the few examples whereby it was possible to devise a drug for TTR amyloidogenesis [23] that acts through the stabilization of a native structure, thus indicating that thermodynamics studies and modeling of tetramer dissociation are a very important target.

To mimic the pathological situation, previous site-directed mutagenesis, namely, mutation F87M/L110M (MT-TTR), was carried out to promote the dissociation of TTR tetramer into monomers [5]. To further shift the equilibrium towards monomers to obtain a more homogeneous dissociated species, an additional amino acid replacement has been introduced in MT-TTR molecule (S117E) [24]. Indeed, the latter mutant containing three mutations (3M-TTR) proved to be markedly more prone to* in vitro* monomerization in comparison with the double mutant MT-TTR, whose tetrameric states could be recovered by Tafamidis binding, at variance with 3M-TTR [24].

The three mutations are not reported in the ClinVar database [25], although pathological mutations do occur in neighbouring residues.

### 1.5. TTR Amyloidosis Therapeutics

As mentioned previously, the dissociation of TTR tetramer is believed to be the initial step into its fibrillation pathway [26]. Therefore, many strategies to TTR amyloidosis prevention exploit its ability to bind small molecules in the T4 binding channel (see Figure 1(a)), mimicking its hormone binding capability, thereby producing kinetic stabilization of the tetramer [27, 28]. Other clinical remedies to FAC and FAP amyloidosis often employ organs transplantation (e.g., liver), even though not all the affected organs can be transplanted (e.g., choroids plexus, where TTR is produced as well [29]). Furthermore, additional treatments against TTR amyloidosis have been experimented, among which there are gene therapy [30] and the resorption of amyloid deposits [31].

The aim of this work is to provide a rationale of the observed dynamics and thermodynamics of the wild-type and mutant transthyretin based on atomistic molecular dynamics simulations. The methods applied here show the relative stability of monomers, dimers, and tetramers and provide a description of both enthalpic and entropic contributions taking advantage of recently developed methods.

## 2. Materials and Methods

### 2.1. Free Energy from End-Point MD Simulation

The free energy of a system, with respect to some reference state, can be in principle deduced from conformational samples obtained, e.g., in a molecular dynamics simulation.

For a well equilibrated system, the enthalpy can be estimated as an ensemble average of the potential energy over a set of conformations from the MD trajectory. The bottleneck in obtaining the Gibbs free energy is the proper modeling of the entropic part and in particular of the solvation entropic effects.

In order to obtain free energy estimates all molecular dynamics snapshots have been analyzed using the GBSA implicit solvent model as implemented in a home-written version of the software Bluues [32, 33].

The Gibbs free energy in the context of implicit solvent MD simulation has been discussed in reviews [34–37]. We follow here our recent perspective on the issue [37]. The free energy of a system may be written as

(1) ΔGA0=−kBTlog⁡∫exp⁡−βUrA+ΔWrA,TdrA

where *U* is the solute potential, *U* + Δ*𝒲* is the potential of mean forces (with Δ*𝒲* the solvation energy), *k*_B_ is the Boltzmann constant, and *T* is the temperature.

The above expression can be rearranged and written as the sum of an entropic term involving only solute coordinates and an ensemble average including also solvent enthalpy and entropy:

(2) $$\begin{array}{c} \Delta G_{A}^{0}=\langle \;U\left(\;r_{A}\;\right)+\Delta W\;\rangle -T\Delta S^{conf} \end{array}$$

where Δ*S*^*conf*^ is the configurational entropy of the solute.

The above formula constitutes the basis of free energy estimation from end-point simulations detailed in the following sections. The solute vacuum potential energy was obtained by selecting the solute from the trajectories and recalculating the energy involving only solute terms.

The solute potential energy and the solvation energy were calculated from molecular dynamics snapshots of the solute. First the molecular surface was generated using the program MSMS by Sanner [38]; then the vertices and normals to the surface were read by the program Bluues [32, 33] and used to compute Generalized Born radii and to compute solvation energy according to the GB model [39].

The solvent accessible surface outputted by MSMS was used to compute the apolar contribution to the solvation energy using a surface tension constant of 5 cal/(Å^2^ mol) [40, 41].

For entropic contributions Δ*S*^*conf*^ is rewritten in terms of solute conformational probability density:

(3) $$\begin{array}{c} \Delta S^{conf}=-R<\log\left(\;\rho \left(\;r_{A},T\;\right)\;\right)> \end{array}$$

where *ρ*(**r**_*A*_, *T*) is the density in configurational space. The latter is estimated using the nearest neighbour method [42–50]. The basic idea of the method is to provide a description of the configuration in a space where a metric is defined (e.g., the torsional angle space) and then use the distance to the *k*^*th*^ nearest neighbour of each sample to estimate the density of probability around that sample. When this is done with the caveats discussed in depth by Demchuk and coworkers [42], the average of the estimated density at each sample provides the estimate of the configurational entropy by the equation above.

In order to cope with the high dimensionality of the space, with largely decoupled degrees of freedom, the mutual information expansion (MIE) method [44, 45] and the maximum information spanning tree method (MIST) [51, 52] were proposed.

The reader is referred to the cited literature for details on the methods.

Entropic contributions due to changes in the internal degrees of freedom were calculated using the nearest neighbour and the MIST methods as implemented in our program PDB2ENTROPY (URL: https://github.com/federico-fogolari/pdb2entropy) which will be described elsewhere.

Rotational and orientational entropy was computed using the nearest neighbour method in the Euclidean space approximation, which is an excellent approximation for the number of samples at hand, as described previously [50]. The calculation is implemented in our program PDB2TRENT (URL: https://github.com/federico-fogolari/pdb2trent) which will be described elsewhere. The same approach has been used by us before [53].

### 2.2. Molecular Models

The X-ray structures used as starting configuration are the wild-type transthyretin (WT-TTR) taken from the RCSB Protein Data Bank (PDB) [54], PDB id: 1F41 [9], solved at the resolution of 1.3 Å; the double point mutant (MT-TTR) F87M/L110M (PDB id: 1GKO [5]) resolved at 2.10 Å; and the in silico engineered triple mutant F87M/L110M/S117E (3M-TTR). The structure of triple mutant was obtained from that of MT-TTR mutating the S117 by E (S117E) using the protein modeling software Swiss-PdbViewer [55]. The N-terminal residues 1-9 and C-terminals 126-127 are not present in the structure and were not modeled. Tetramers were built from deposited asymmetric units (dimers) by applying crystal symmetry operations. All the crystallization water molecules were removed prior to run the simulations.

### 2.3. Molecular Dynamics Simulations

A set of independent simulation runs of different length ranging from 50 to 250 ns were performed on monomer, dimer, and tetramer of wild-type and mutant structures of TTR employing either CHARMM27 all atoms force field with CMAP correction [56] or amber99sb-ildn molecular mechanics force field [57]. The protein atoms were placed at the center of a cubic box; the system was solvated using the 3-site rigid water model TIP3P [58–60]. In each system an equivalent number of solvent molecules were replaced by Na^+^ counterions to obtain a neutral system.

The systems were first minimized using the steepest descent minimization algorithm with a minimization step size of 0.1 nm and a maximum convergence force of 1000.0 kJmol^−1^nm^−1^. The equilibration phase was done in 2 steps: 100 ps in* NVT* ensemble followed by 100 ps in* NPT* ensemble. During the first equilibration stage, the leap-frog integrator with integration time-step of 0.002 ps was used to update the changes in the system. Particle Mesh Ewald summation [61, 62] accounted for long-range electrostatics interactions. The temperature was equilibrated to a reference value of 300 K using the velocity rescaling (modified Berendsen thermostat) [63] with a coupling constant of 0.1 ps. Short-range electrostatics and van der Waals interactions were truncated with a 10 Å cutoff. All bonds were constrained with the LINCS algorithm [64]. In* NPT* equilibration stage, the previous parameters were still used and the pressure was stabilized to 1.0 bar using the Parrinello-Rahman pressure coupling [65, 66] with a coupling constant of 2.0 ps.

### 2.4. Molecular Dynamics Simulation Analysis

MD trajectories were analyzed with available structural-based tools in* Gromacs*-5.0.4 [41, 67]. The thermodynamic stability of the tetrameric systems was further processed using the Academic License version of the bioinformatics tool* FoldX* [68], using the commands* Stability* and* AnalyseComplex*, respectively, to gain information on protein stability and interacting interface free energies. In all the cases, the values were averaged over the whole simulation trajectory.* AnalyseComplex* command outputs the Gibbs interacting free energy of binding for a complex formation (folding), say AB (*A* + *B* → *AB*), computed as ΔΔ*G*_AB_ = Δ*G*_AB_ − (Δ*G*_A_ + Δ*G*_B_), where Δ*G* is the free energy of folding. Similarly the statistical effective energy function proposed by Berrera et al. [69], referred hereafter as* BMF*, has been used with home-written routines.

The computation of conformational entropy employs the nearest neighbour formalism whose rationale is the estimation of the local probability density around each sample by counting its number of neighbours within a hypersphere of radius equal the distance from that sample to its *k*^th^ nearest neighbours. Thus, the values discussed in the results section are averaged for the 10^th^ nearest neighbour, set as default for the routines previously cited. Conformational entropies were calculated over 5000 frames.

Pictures were either collected with PyMOL [70] or VMD [71], secondary structures were assigned using DSSP program [72], and H-bonds occupancy was computed using the readHBmap.py tool reporting only H-bonds with occupancy greater than 10 %.

## 3. Results and Discussion

### 3.1. Monomer to Dimer and Dimer to Tetramer Associations

The enthalpic contributions to the association of monomers and dimers were assessed considering both single species simulations and detaching from the tetramer simulations dimers first and then monomers. In both cases favorable protein-protein interactions appear largely overestimated by the force-field used, as recently observed also for other systems [73, 74]. The “enthalpic” contributions encompass the solvation energy which includes also the entropy of the solvent. For this reason the term enthalpy is used rather freely only with the purpose of separating the terms related to the potential of mean force and those computed from the analysis of conformational samples.

The enthalpy of two wild-type (mutant) monomers associating in a dimer computed by the ensemble averages of the potential is -49.7 (-49.8) kcal/mol and that of two dimers associating in a tetramer is -81.0 (-77.2) kcal/mol leading to a very favorable -180.4 (-176.8) kcal/mol association enthalpy for tetramerization.

The reduction in conformational entropy (i.e., changes in entropy due to internal degrees of freedom) from two monomers to dimer and from two dimers to tetramer, computed considering only correlations within each residue, is -6.7 (-12.8) e.u. and -27.2 (-24.8) e.u., respectively, thus opposing tetramerization, at 300K, by 24.3 (30.1) kcal/mol.

The rotational-translational entropy (Δ*S*^*rt*^) was computed for monomer-monomer and dimer-dimer association, using the Euclidean approximation for the rotational-translational space, resulting in 19.0 (17.3) e.u. for the dimer-dimer association and 17.5 (17.5) e.u. for the monomer-monomer association.

Rotational-translational entropy thus opposes tetramerization by 32.2 (31.2) kcal/mol.

Overall, therefore, the large favorable enthalpy (likely to be overestimated here) is opposed by conformational and rotational-translational entropy which we can estimate to result in total 56.5 (61.3) kcal/mol. These results are summarized in Table 1.

The detailed analysis of the terms contributing to the enthalpy shows that covalent energy terms contribute very little to the energy difference and Lennard-Jones terms make the largest favorable contribution to association, whereas favorable electrostatic and unfavorable desolvation contributions provide an overall unfavorable but smaller (about one-third in absolute value) contribution.

The analysis provides thus a lower stability of the tetramer assembly for the mutant relative to the wild-type protein. The uncertainties of the methodology are however comparable to this difference.

### 3.2. Statistical Effective Energy Functions Analysis

**Figure 3:**
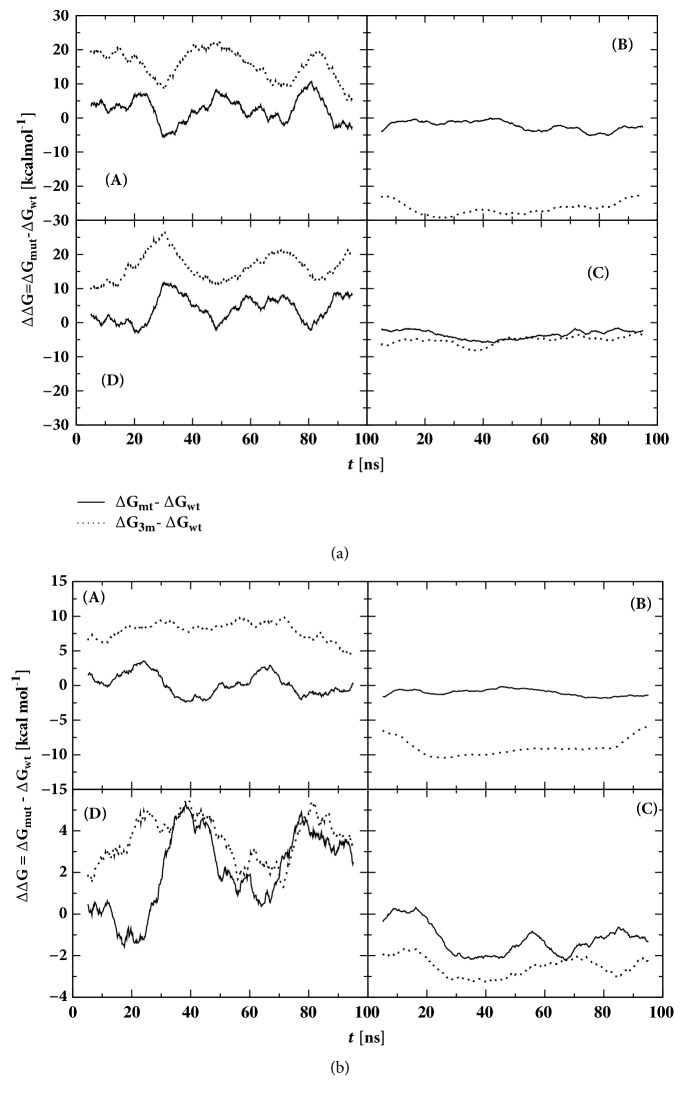
Folding free energy difference between mutants and native wild-type along the simulation trajectory and throughout the tetramer-unfolded monomer equilibrium. Panels (a) and (b) refer to* FoldX* and* BMF* results, respectively. In the case of tetramer (A), ΔΔG_T_ is the global stability free energy change of tetramers, i.e., the free energy required to fold the tetramers from their unfolded monomers. Subscripts *mut* and *wt* are, respectively, mutant (MT-TTR or 3M-TTR, with their simplified notations *mt* and* 3m*) and wild-type (WT-TTR). ΔΔG_D_ (B) is the free energy necessary to form the dimers from tetramers (T→D). ΔΔG_M_ (C) is the free energy involved in the dissociation of dimers into folded monomers (D→M) and ΔΔG_M_^unf^ (D) is the free energy of unfolding of individual monomers (M →M^unf^).

The structural and thermodynamic stability of both mutant structures in relation to wild-type was further probed thanks to two independent bioinformatic tools, i.e.,* FoldX* [68] (Figure 3(a)) and the statistical effective energy function* BMF* [69] (Figure 3(b)). This analysis provides a complementary view of the thermodynamic picture presented above.

We computed the folding free energy differences between mutants and wild-type (ΔΔG=ΔG_mutant_-ΔG_wild-type_) throughout the tetramer-unfolded monomers equilibrium, Figure 3. Some words of caution are due when presenting the following data because the energy functions used take into account implicitly the entropy loss from internal degrees of freedom but not that arising from external degrees of freedom as implied by the tetramer to dimers and the dimers to monomers transitions.

Finally, notwithstanding the fluctuations and differences in underlying principles and datasets, the two approaches used here are consistent with each other as detailed below.

The panels (A) in Figures 3(a) and 3(b) point out that both mutants have positive difference in free energy as compared to WT-TTR and thus are less stable. Their average free energy ΔΔG_T_ computed using* BMF* (*FoldX*) is +0.3 and +7.8 kcal/mol (+2.5 and +15.6 kcal/mol), respectively, for MT-TTR and 3M-TTR, confirming the decreased tetramer thermodynamic stability upon mutations, significant in the case of 3M-TTR. This result is consistent with previously determined experimental data, which indicated that monomers of 3M-TTR, at variance with MT-TTR, could not reform tetramers upon the addition of Tafamidis, a known stabilizer of the tetrameric form [24].

In order to track down all contributions, ΔΔG's values were computed and reported in subsets (B, C, and D) of Figures 3(a) and 3(b) for all the steps in the equilibrium pathway from tetramers to unfolded monomers (required before monomers self-assemble into amyloid fibrils) through dimers. We called ΔΔG_D_, ΔΔG_M_, and ΔΔG_M_^unf^ the free energy for the dissociation of tetramers into dimers (T→D), for the formation of folded monomers from dimers (D→M), and for the unfolding of individual monomers (M→M^unf^), respectively. Based on simulations we guessed that tetramers first break down into dimers I/II and III/IV, i.e., along the C_2_ crystallographic axis, instead of I/III and II/IV. Thus, ΔΔ*G*_D_ = Δ*G*_D_ − Δ*G*_T_ (dissociation free energy at I/II-III/IV dimer-dimer contact), ΔΔ*G*_M_ = Δ*G*_M_ − Δ*G*_D_ (dissociation free energy at I-II and III-IV monomer-monomer contacts), and ΔΔ*G*_M_^unf^ = Δ*G*_M_^unf^ − Δ*G*_M_.

ΔΔG_D_ using* BMF* (*FoldX*) average is -1.1 and -8.7 kcal/mol (-2.5 and -26.3 kcal/mol) for MT-TTR and 3M-TTR, respectively, relative to WT-TTR. This means both equilibriums are shifted towards the right (subsets (B) in Figures 3(a) and 3(b)), i.e., the formation of dimers. The quite high (absolute) value displayed by 3M-TTR indicates the propensity of tetrameric assembly of the latter structure to dissociate into dimers I/II and III/IV as compared to the wild-type protein. Considering the dimer-monomer equilibrium (subsets (C) in Figures 3(a) and 3(b)), ΔΔG_M_'s average using* BMF* (*FoldX*) is -1.2 and -2.5 kcal/mol (-3.3 and -5.2 kcal/mol) over the trajectory for MT-TTR and 3M-TTR, respectively, relative to WT-TTR. The values are however rather limited compared to the overall stability computed using* BMF* for the process of four WT-TTR monomers associating into 2 dimers (-14.2 kcal/mol).

Finally the change with respect to WT-TTR in folding free energy of monomers has been computed. Both* BMF* and* FoldX* predict a shift towards the formation of more stable monomers for both mutants ΔΔG_M_^unf^> 0; i.e., the equilibrium M⇄M^*unf*^ is shifted towards the folded form (panels (D) in Figures 3(a) and 3(b)). The unfavorable formation of unfolded monomers in both mutants strongly correlates with the secondary structure analysis displayed in Figure 5 showing that notwithstanding the enhanced dissociation of tetramers into monomers through dimers monomers remain stable (folded). Besides, this result would strongly suggest that both monomers of our mutant variants are nonamyloidogenic, as is known as far as MT-TTR is concerned [5].

### 3.3. Localised Structural Transitions

**Figure 4:**
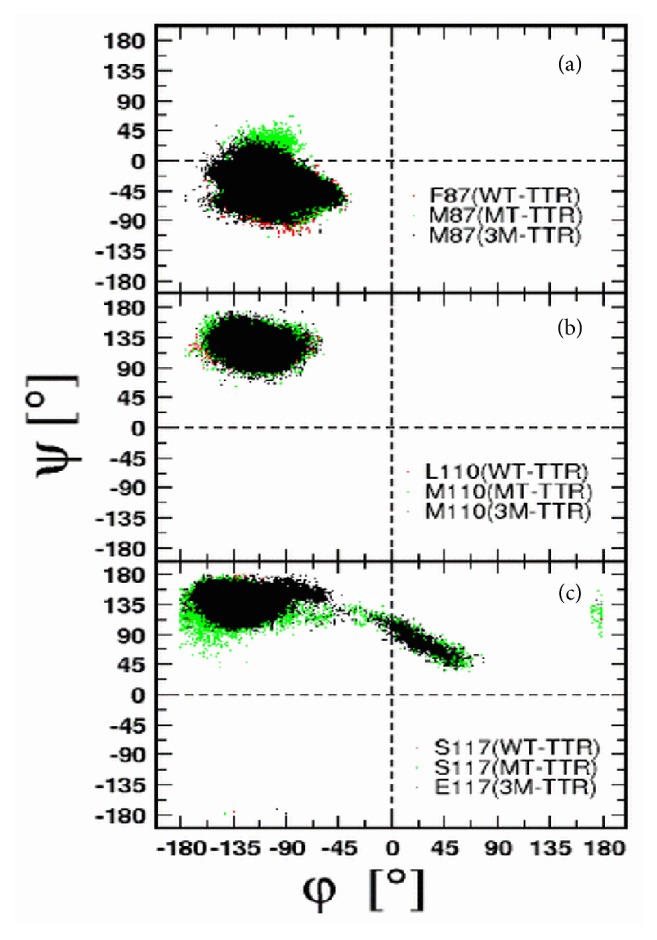
Ramachandran plots of individual residues at individual point mutations.

Local conformational transitions were assessed by computing the dihedral phi (*ϕ*) and psi (*ψ*) angles of individual residues involved in the mutation and represented as Ramachandran plots, Figure 4.

The X-ray (*ϕ*°, *ψ*°) dihedral angles for residue 87 are (-84,-55), (-84,-40), and (-85,-41) in WT-TTR, MT-TTR, and 3M-TTR, respectively. The same values reported from simulation are as follows: (-75±12, -46±9), (-80±16, -44±13), and (-91±15, -38±15), Figure 4(a). These latter lie in the allowed regions for *α*-helical secondary structure, −89 < *ϕ* < −39 and −66 < *ψ* < −16 [75], confirming that no secondary structural transition occurred at this position.

The *ϕ*/*ψ* dihedral angles of L110 and M110 residues Figure 4(b) look even more similar than the previously observed ones. No major deviation was observed in individual monomers and the dihedral angles for residue 110 in each variant keep close to the equilibrium value. The average reported simulation values are (-122±11, 122±8), (-116±12, 122±10), and (-117±11, 119±10), respectively, in WT-TTR, MT-TTR, and 3M-TTR. Indeed, these fall into the region corresponding to *β*-sheet secondary structure, −180 < *ϕ* < −45 and 45 < *ψ* < 225 [75], and imply that no structural transition is seen in the process of point mutation L110M.

In Figure 4(c), while both TTR variants are displaying nearly the same *ϕ*/*ψ* averages, (-137±10, 140±9), (-136±16, 134±12), and (-131±17, 135±11), it is seen that a distinct region is being accessed by mutant variants. The region is defined by −40 < *ϕ* < 70 and 30 < *ψ* < 140 corresponding to left handed helix conformation. The transition is observed only once for both MT-TTR and 3M-TTR within the first 10 ns of the simulation possibly as a transient adaptation to in silico mutation of residue 110.

### 3.4. Secondary Structural Changes

**Figure 5:**
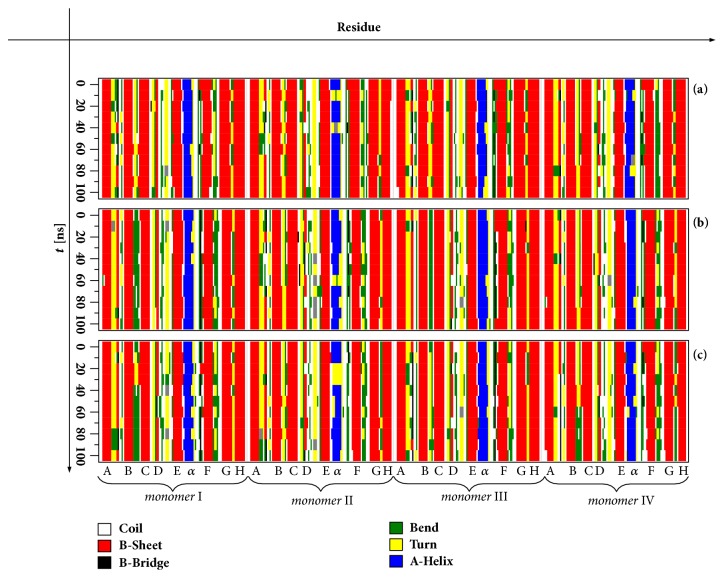
Time-based secondary structure analyses. From top to bottom the secondary structures of WT-TTR (a), MT-TTR (b), and 3M-TTR (c) are, respectively, displayed. Colours were used to distinguish between secondary structure types and letters A to H stand for *β*-strands and *α* is the helical portion between E and F strands.

The illustration of dynamical processes in our systems during the simulations was done by computing the secondary structure, Figure 5. The latter analysis was performed thanks to the DSSP program which determines the existence of hydrogen bonds as a criterion for the presence of secondary structure [72]. While dihedral (*ϕ*, *ψ*) angles (Figure 4) were useful for assessment of local structural changes, secondary structure is able to highlight the global structural changes.

In Figure 5 we can see that the helical region exhibits large structural fluctuations and even gets disrupted from time to time, like in monomer II for all the TTR variants. F-strand undergoes some structural fluctuations at its beginning, particularly in monomers I, III, and IV. In G-H loop, some part of the structure is being converted from bend to *β*-turn and conversely. D-strand displays quite high structural fluctuations in monomers II and IV of WT-TTR, in monomers II and III of MT-TTR, and in all monomers for 3M-TTR. A- and C-strands make the most stable parts of the molecules. E-strand in the vicinity of *α*-helix is showing some fluctuations in monomers I, III, and IV of all the variants. In spite of structural fluctuations exhibited by major parts of the tetrameric molecular assemblies, Figure 5 clearly points out that monomers are nevertheless remaining folded; i.e., they preserve essentially their secondary structures. This observation is supported by the fact that no evident disruption and secondary structure conversion are seen in the core region (made by the *β*-strands) of the different systems studied.

### 3.5. Intra- and Interchains Hydrogen Bonds Occupancy

**Figure 6:**
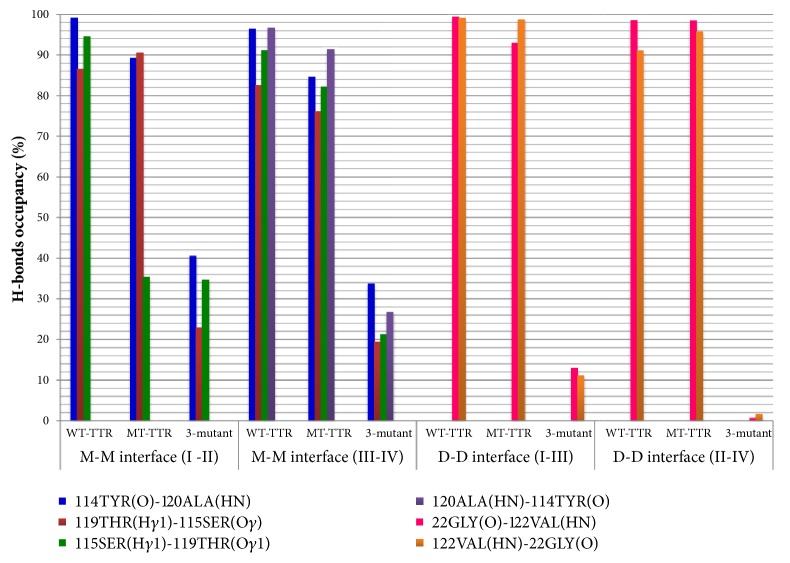
Summary of H-bonds with their occupancies identified to destabilize the tetrameric structure of TTR. M and D stand, respectively, for monomer and dimer while I to IV are different monomers.

In order to understand the mechanism of 3M-TTR dissociation we computed the H-bonds occupancy along the MD trajectory considering both main chain-main chain, main chain-side chain, and side chain-side chain hydrogen bonds, at monomer-monomer and dimer-dimer interfaces.  Figure 6 summarizes the occupancies of the interactions that were identified to significantly perturb the tetrameric assembly of TTR upon mutations. It should be noted that for 3M-TTR the occupancy reflects an average of both (starting) associated and dissociated configurations.

In Figure 6, seven major H-bonds (with ⩾ 80 % occupancy) can be seen almost symmetrically distributed in both dimers, three at I-II interface and four at III-IV interface. These are being lost in 3M-TTR with their average occupancy dropping below 40 %. These include the interactions Y114(CO)-A120(HN) and A120(HN)-Y114(O) (main chain-main chain) and T119(H^*γ*1^)-S115(O^*γ*^) and S115(H^*γ*1^)-T119(O^*γ*1^) (side chain-side chain). Most of them are located in the H-strand and some in the G-H loop.

At the interface of symmetric units (I-III and II-IV) there is a significant loss in H-bond occupancy in 3M-TTR, in particular at II-IV interface. The occupancies of 22GLY(O)-122VAL(HN) and 122VAL(HN)-22GLY(O) (main chain-main chain) located in H-strand and A-B loops lie under the 10 % (Figure 6). Interestingly, it is worth mentioning that, in 3M-TTR, several side chain-side chain interactions are being formed following dissociation and subsequent temporary association. These involve many inner sheets residues like K15, R104, and E117, most of which show significant deviation from the starting WT-TTR X-ray structure.

Analysis of hydrogen bonds connectivity confirms the pivotal role of such networks in preserving the protein integrity, typically in some dedicated portions, in this case H-strand, G-H, and A-B loops. These susceptible portions of TTR that build up the monomer-monomer and dimer-dimer interfaces are definitely playing a key role in destabilization of tetrameric subunits of TTR, especially in the case of 3M-TTR. Indeed, the disappearance of H-bonds in the previously mentioned domains along the timescale of the simulations brings a possible explanation on the integrity loss of tetramers in 3M-TTR.

### 3.6. Mechanism of Tetramer Dissociation

**Figure 7:**
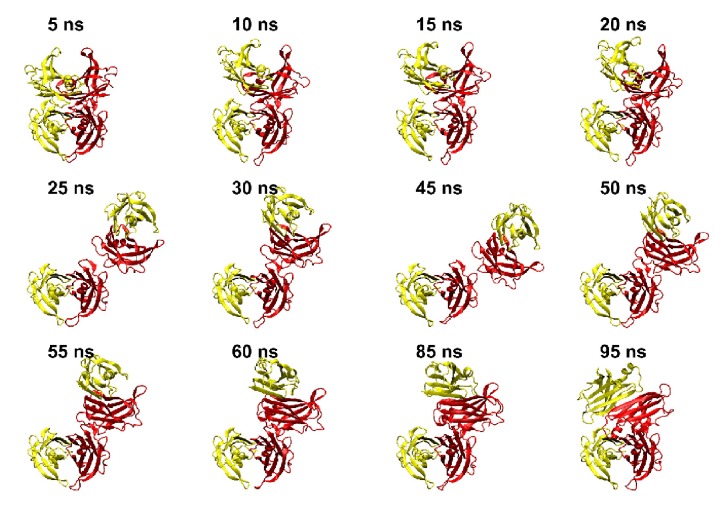
Snapshots from 3M-TTR 100 ns molecular dynamics simulations. Chains I and III are shown in red; chains II and IV are shown in yellow. Chains I and II, in the lower part of the tetramer, are used for superposing all snapshots on the starting conformation.

The path of dissociation of dimers in 3M-TTR may be followed during the simulation (Figure 7). The first step in conformational transition is the dissociation at the interface I/IV involving residues 17-24 and 110-123, including position 117 mutated to glutamic acid in 3M-TTR at variance with MT-TTR where the corresponding residue is a serine. The transition appears driven by the electrostatic repulsion of the pairs of acidic residues E117 close in each dimer. First the interface is weakened (up to 15 ns) and disrupted (20 ns); then both I/II and III/IV dimers remain rigid. Dimer III/IV rotates for most of the simulation about a hinge centered on salt bridges E51 (I)-R104 (III) and R104 (I)-E51 (III) and hydrogen bonds E51 (III)-T123 (I) and at the end only about the latter two interactions. The final (possibly transient) conformation is stabilized by salt bridges E117 (I)-R21 (III) and K15-E54 at I/III interface. Other interactions at II/III interface are mostly hydrophobic. No sign of dissociation or conformational rearrangement at dimers is observed during the simulation. For both dimers the proximity of E117 acidic groups should result in repulsion which is however reduced by ionic interactions.

## 4. Conclusions

Molecular dynamics simulations were used in this work to probe the structural stability and the dynamics of engineered mutants of transthyretin (TTR), i.e., the double mutant F87M/L110M (MT-TTR) and the triple mutant F87M/L110M/S117E (3M-TTR), in relation to wild-type. The analysis of trajectories reveals that mutations do not have major impact on protein structure, and the thermodynamic analysis confirms this picture.

Estimation of the free energy from end-point simulations shows that the two mutations F87M/L110M on monomers/dimer and dimers/tetramer equilibria favor the dissociation. The results are however within the uncertainties of the methodology.

Consistent with the latter analysis evaluation of stability with the statistical effective energy functions* FoldX* and* BMF* shows that the mutations and the triple mutations F87M/L110M/S117E affect differently the same equilibria and the stability of the monomers. The latter is almost not perturbed by mutations, whereas the equilibria are shifted towards the dissociated species relative to wild-type TTR. This is confirmed by the analysis of 3M-TTR which shows partial dissociation within the first 20 ns of the simulation, implying that contacts are lost at the dimer-dimer interface, whereas dimers appear fairly stable.

Overall the simulations provide a detailed view of the dynamics and thermodynamics of wild-type and mutant transthyretin and a rationale of the observed effects.

## Figures and Tables

**Table 1:** Summary of potential and solvation energy average and conformational entropy changes computed using the nearest neighbour approach from monomer (M) to dimer (D) and from dimer to tetramer (T).

		Δ*H* (kcal mol^−1^)	-TΔ*S*^conf^ (kcal mol^−1^)	-TΔ*S*^rt^ (kcal mol^−1^)
WT-TTR	2M→D	-49.7 ± 3.7	4.0 ± 0.8	10.4
	2D→T	-81.0 ± 2.9	16.2 ± 0.5	11.3

MT-TTR	2M→D	-49.8 ± 2.6	7.6 ± 0.8	10.4
	2D→T	-77.2 ± 6.5	14.8 ± 1.1	10.3

## Data Availability

The data used to support the findings of this study are available from the corresponding author upon request.

## References

[1] Hamilton J. A., Benson M. D. (2001). Transthyretin: A review from a structural perspective. *Cellular and Molecular Life Sciences*.

[2] Rodrigues J. R., Simões C. J. V., Silva C. G., Brito R. M. M. (2010). Potentially amyloidogenic conformational intermediates populate the unfolding landscape of transthyretin: Insights from molecular dynamics simulations. *Protein Science*.

[3] Ferreira P., Sant'Anna O., Varejão N. (2013). Correction: Structure-Based Analysis of A19D, a Variant of Transthyretin Involved in Familial Amyloid Cardiomyopathy. *PLoS ONE*.

[4] Reinés J. B., Vera T. R., Martín M. U. (2014). Epidemiology of transthyretin-associated familial amyloid polyneuropathy in the Majorcan area: Son Llàtzer Hospital descriptive study. *Orphanet Journal of Rare Diseases*.

[5] Jiang X., Smith C. S., Petrassi H. M. (2001). An engineered transthyretin monomer that is nonamyloidogenic, unless it is partially denatured. *Biochemistry*.

[6] Lim K. H., Dyson H. J., Kelly J. W., Wright P. E. (2013). Localized structural fluctuations promote amyloidogenic conformations in transthyretin. *Journal of Molecular Biology*.

[7] Banerjee A., Bairagya H. R., Mukhopadhyay B. P., Nandi T. K., Mishra D. K. (2013). Conserved water mediated H-bonding dynamics of Ser117 and Thr119 residues in human transthyretinthyroxin complexation: Inhibitor modeling study through docking and molecular dynamics simulation. *Journal of Molecular Graphics and Modelling*.

[8] Blake C. C. F., Geisow M. J., Oatley S. J., Rérat B., Rérat C. (1978). Structure of prealbumin: Secondary, tertiary and quaternary interactions determined by Fourier refinement at 1.8 Å. *Journal of Molecular Biology*.

[9] Hörnberg A., Eneqvist T., Olofsson A., Lundgren E., Sauer-Eriksson A. E. (2000). A comparative analysis of 23 structures of the amyloidogenic protein transthyretin. *Journal of Molecular Biology*.

[10] Haupt M., Blakeley M. P., Fisher S. J. (2014). Binding site asymmetry in human transthyretin: Insights from a joint neutron and X-ray crystallographic analysis using perdeuterated protein. *IUCrJ*.

[11] Cianci M., Folli C., Zonta F., Florio P., Berni R., Zanotti G. (2015). Structural evidence for asymmetric ligand binding to transthyretin. *Acta Crystallographica Section D: Biological Crystallography*.

[12] Dubrey S., Ackermann E., Gillmore J. (2015). The transthyretin amyloidoses: Advances in therapy. *Postgraduate Medical Journal*.

[13] Pasquato N., Berni R., Folli C., Alfieri B., Cendron L., Zanotti G. (2007). Acidic pH-induced Conformational Changes in Amyloidogenic Mutant Transthyretin. *Journal of Molecular Biology*.

[14] Lai Z., Colón W., Kelly J. W. (1996). The acid-mediated denaturation pathway of transthyretin yields a conformational intermediate that can self-assemble into amyloid. *Biochemistry*.

[15] Chiti F., Dobson C. M. (2006). Protein misfolding, functional amyloid, and human disease. *Annual Review of Biochemistry*.

[16] Invernizzi G., Papaleo E., Sabate R., Ventura S. (2012). Protein aggregation: Mechanisms and functional consequences. *The International Journal of Biochemistry & Cell Biology*.

[17] Chiti F., Dobson C. M. (2017). Protein misfolding, amyloid formation, and human disease: A summary of progress over the last decade. *Annual Review of Biochemistry*.

[18] Greene M. J., Klimtchuk E. S., Seldin D. C., Berk J. L., Connors L. H. (2015). Cooperative stabilization of transthyretin by clusterin and diflunisal. *Biochemistry*.

[19] Lashuel H. A., Lai Z., Kelly J. W. (1998). Characterization of the transthyretin acid denaturation pathways by analytical ultracentrifugation: Implications for wild-type, V30M, and L55P amyloid fibril formation. *Biochemistry*.

[20] Quintas A., Saraiva M. J. M., Britot R. M. M. (1999). The tetrameric protein transthyretin dissociates to a non-native monomer in solution. A novel model for amyloidogenesis. *The Journal of Biological Chemistry*.

[21] Quintas A., Vaz D. C., Cardoso I., Saraiva M. J. M., Brito R. M. M. (2001). Tetramer Dissociation and Monomer Partial Unfolding Precedes Protofibril Formation in Amyloidogenic Transthyretin Variants. *The Journal of Biological Chemistry*.

[22] Jiang X., Buxbaum J. N., Kelly J. W. (2001). The V122I cardiomyopathy variant of transthyretin increases the velocity of rate-limiting tetramer dissociation, resulting in accelerated amyloidosis. *Proceedings of the National Acadamy of Sciences of the United States of America*.

[23] Johnson S. M., Connelly S., Fearns C., Powers E. T., Kelly J. W. (2012). The transthyretin amyloidoses: From delineating the molecular mechanism of aggregation linked to pathology to a regulatory-agency-approved drug. *Journal of Molecular Biology*.

[24] Zanotti G., Vallese F., Ferrari A. (2017). Structural and dynamics evidence for scaffold asymmetric flexibility of the human transthyretin tetramer. *PLoS ONE*.

[25] Landrum M. J., Lee J. M., Benson M. (2018). ClinVar: improving access to variant interpretations and supporting evidence. *Nucleic Acids Research*.

[26] Kelly J. W., Lansbury P. T. (1994). A chemical approach to elucidate tin mechanism of transthyretin and *β*protein amyloid fibril formation. *Amyloid*.

[27] Peterson S. A., Klabunde T., Lashuel H. A., Purkey H., Sacchettini J. C., Kelly J. W. (1998). Inhibiting transthyretin conformational changes that lead to amyloid fibril formation. *Proceedings of the National Acadamy of Sciences of the United States of America*.

[28] Almeida M. R., Gales L., Damas A. M., Cardoso I., Saraiva M. J. (2005). Small transthyretin (TTR) ligands as possible therapeutic agents in TTR amyloidoses. *CNS & Neurological Disorders - Drug Targets *.

[29] Ando Y., Ueda M. (2012). Diagnosis and therapeutic approaches to transthyretin amyloidosis. *Current Medicinal Chemistry*.

[30] Nakamura M., Ando Y. (2004). Applications of gene therapy for familial amyloidotic polyneuropathy. *Expert Opinion on Biological Therapy*.

[31] Sebastião M. P., Merlini G., Saraiva M. J., Damas A. M. (2000). The molecular interaction of 4’-iodo-4’-deoxydoxorubicin with Leu-55Pro transthyretin ‘amyloid-like’ oligomer leading to disaggregation. *Biochemical Journal*.

[32] Fogolari F., Corazza A., Yarra V., Jalaru A., Viglino P., Esposito G. (2012). Bluues: A program for the analysis of the electrostatic properties of proteins based on generalized Born radii. *BMC Bioinformatics*.

[33] Walsh I., Minervini G., Corazza A., Esposito G., Tosatto S. C. E., Fogolari F. (2012). Bluues server: Electrostatic properties of wild-type and mutated protein structures. *Bioinformatics*.

[34] Gilson M. K., Given J. A., Bush B. L., McCammon J. A. (1997). The statistical-thermodynamic basis for computation of binding affinities: A critical review. *Biophysical Journal*.

[35] Roux B., Simonson T. (1999). Implicit solvent models. *Biophysical Chemistry*.

[36] Wereszczynski J., McCammon J. A. (2012). Statistical mechanics and molecular dynamics in evaluating thermodynamic properties of biomolecular recognition. *Quarterly Reviews of Biophysics*.

[37] Fogolari F., Corazza A., Esposito G. (2018). Free Energy, Enthalpy and Entropy from Implicit Solvent End-Point Simulations. *Frontiers in Molecular Biosciences*.

[38] Sanner M. F., Olson A. J., Spehner J.-C. (1996). Reduced surface: An efficient way to compute molecular surfaces. *Biopolymers*.

[39] Bashford D., Case D. A. (2000). Generalized born models of macromolecular solvation effects. *Annual Review of Physical Chemistry*.

[40] Sitkoff D., Sharp K. A., Honig B. (1994). Accurate calculation of hydration free energies using macroscopic solvent models. *The Journal of Physical Chemistry C*.

[41] Abraham M. J., van der Spoel D., Lindahl E., Hess B. (2014). *the GROMACS development team: GROMACS User Manual version 5.0.4*.

[42] Singh H., Misra N., Hnizdo V., Fedorowicz A., Demchuk E. (2003). Nearest neighbor estimates of entropy. *American Journal of Mathematical and Management Sciences*.

[43] Hnizdo V., Fedorowicz A., Singh H., Demchuk E. (2003). Statistical thermodynamics of internal rotation in a hindering potential of mean force obtained from computer simulations. *Journal of Computational Chemistry*.

[44] Killian B. J., Yundenfreund Kravitz J., Gilson M. K. (2007). Extraction of configurational entropy from molecular simulations via an expansion approximation. *The Journal of Chemical Physics*.

[45] Hnizdo V., Jun T., Killian B. J., Gilson M. K. (2008). Efficient calculation of configurational entropy from molecular simulations by combining the mutual-information expansion and nearest-neighbor methods. *Journal of Computational Chemistry*.

[46] Fenley A. T., Killian B. J., Hnizdo V., Fedorowicz A., Sharp D. S., Gilson M. K. (2014). Correlation as a determinant of configurational entropy in supramolecular and protein systems. *The Journal of Physical Chemistry B*.

[47] Huggins D. J. (2014). Comparing distance metrics for rotation using the k-nearest neighbors algorithm for entropy estimation. *Journal of Computational Chemistry*.

[48] Huggins D. J. (2015). Quantifying the entropy of binding for water molecules in protein cavities by computing correlations. *Biophysical Journal*.

[49] Fogolari F., Corazza A., Fortuna S. (2015). Distance-based configurational entropy of proteins from molecular dynamics simulations. *PLoS ONE*.

[50] Fogolari F., Dongmo Foumthuim C. J., Fortuna S., Soler M. A., Corazza A., Esposito G. (2016). Accurate Estimation of the Entropy of Rotation-Translation Probability Distributions. *Journal of Chemical Theory and Computation*.

[51] King B. M., Tidor B. (2009). MIST: Maximum Information Spanning Trees for dimension reduction of biological data sets. *Bioinformatics*.

[52] King B. M., Silver N. W., Tidor B. (2012). Efficient calculation of molecular configurational entropies using an information theoretic approximation. *The Journal of Physical Chemistry B*.

[53] Dongmo Foumthuim C. J., Corazza A., Esposito G., Fogolari F. (2017). Molecular dynamics simulations of *β*2-microglobulin interaction with hydrophobic surfaces. *Molecular BioSystems*.

[54] Berman H. M., Westbrook J., Feng Z. (2000). The protein data bank. *Nucleic Acids Research*.

[55] Johansson M. U., Zoete V., Michielin O., Guex N. (2012). Defining and searching for structural motifs using DeepView/Swiss-PdbViewer. *BMC Bioinformatics*.

[56] Lindahl E., Bjelkmar P., Larsson P., Cuendet M. A., Hess B. (2010). Implementation of the charmm force field in GROMACS: Analysis of protein stability effects from correction maps, virtual interaction sites, and water models. *Journal of Chemical Theory and Computation*.

[57] Lindorff-Larsen K., Piana S., Palmo K. (2010). Improved side-chain torsion potentials for the Amber ff99SB protein force field. *Proteins: Structure, Function, and Bioinformatics*.

[58] Jorgensen W. L., Chandrasekhar J., Madura J. D., Impey R. W., Klein M. L. (1983). Comparison of simple potential functions for simulating liquid water. *The Journal of Chemical Physics*.

[59] Jorgensen W. L., Madura J. D. (1985). Temperature and size dependence for monte carlo simulations of TIP4P water. *Molecular Physics*.

[60] Mahoney M. W., Jorgensen W. L. (2000). A five-site model for liquid water and the reproduction of the density anomaly by rigid, nonpolarizable potential functions. *The Journal of Chemical Physics*.

[61] Darden T., York D., Pedersen L. (1993). Particle mesh Ewald: an N·log(N) method for Ewald sums in large systems. *The Journal of Chemical Physics*.

[62] Essmann U., Perera L., Berkowitz M. L., Darden T., Lee H., Pedersen L. G. (1995). A smooth particle mesh Ewald method. *The Journal of Chemical Physics*.

[63] Bussi G., Donadio D., Parrinello M. (2007). Canonical sampling through velocity rescaling. *The Journal of Chemical Physics*.

[64] Hess B., Bekker H., Berendsen H. J. C., Fraaije J. G. E. M. (1997). LINCS: a linear Constraint Solver for molecular simulations. *Journal of Computational Chemistry*.

[65] Parrinello M., Rahman A. (1981). Polymorphic transitions in single crystals: a new molecular dynamics method. *Journal of Applied Physics*.

[66] Nosé S., Klein M. L. (1983). Constant pressure molecular dynamics for molecular systems. *Molecular Physics*.

[67] Hess B., Kutzner C., van der Spoel D., Lindahl E. (2008). GRGMACS 4: algorithms for highly efficient, load-balanced, and scalable molecular simulation. *Journal of Chemical Theory and Computation*.

[68] Schymkowitz J., Borg J., Stricher F., Nys R., Rousseau F., Serrano L. (2005). The FoldX web server: an online force field. *Nucleic Acids Research*.

[69] Berrera M., Molinari H., Fogolari F. (2003). Amino acid empirical contact energy definitions for fold recognition in the space of contact maps. *BMC Bioinformatics*.

[70] Delano W. L. (2002). The PyMOL Molecular Graphics System Version 1.7.3.0 Schrödinger, LLC. http://www.pymol.org/.

[71] Humphrey W., Dalke A., Schulten K. (1996). VMD: visual molecular dynamics. *Journal of Molecular Graphics*.

[72] Kabsch W., Sander C. (1983). Dictionary of protein secondary structure: pattern recognition of hydrogen-bonded and geometrical features. *Biopolymers*.

[73] Petrov D., Zagrovic B. (2014). Are Current Atomistic Force Fields Accurate Enough to Study Proteins in Crowded Environments?. *PLoS Computational Biology*.

[74] Abriata L. A., Dal Peraro M. (2015). Assessing the potential of atomistic molecular dynamics simulations to probe reversible protein-protein recognition and binding. *Scientific Reports*.

[75] Hovmöller S., Zhou T., Ohlson T. (2002). Conformations of amino acids in proteins. *Acta Crystallographica Section D: Biological Crystallography*.

